# BLESS: bagged logistic regression for biomarker identification

**DOI:** 10.3389/fgene.2024.1336891

**Published:** 2024-09-10

**Authors:** Kyle Gardiner, Xuekui Zhang, Li Xing

**Affiliations:** ^1^ Department of Mathematics and Statistics, University of Saskatchewan, Saskatoon, SK, Canada; ^2^ Department of Mathematics and Statistics, University of Victoria, Victoria, BC, Canada

**Keywords:** ensemble learning, genome-wide association study, cognitive function, genomics biomarker, bagging (bootstrap aggregation)

## Abstract

The traditional single nucleotide polymorphism (SNP)-wise approach in genome-wide association studies is focused on examining the marginal association between each SNP with the outcome separately and applying multiple testing adjustments to the resulting *p*-values to reduce false positives. However, the approach suffers a lack of power in identifying biomarkers. We design an ensemble machine learning approach to aggregate results from logistic regression models based on multiple subsamples, which helps to identify biomarkers from high-dimensional genomic data. We use different methods to analyze a genome-wide association study from the Alzheimer’s Disease Neuroimaging Initiative. The SNP-wise approach does not identify any significant signal, while our novel approach provides a list of ranked SNPs associated with the cognitive functions of interests.

## 1 Introduction

Genome-wide association studies (GWASs) are used to identify associations between genetic variations and diseases/traits of interest. A commonly used approach is the SNP-wise approach, which involves surveying single nucleotide polymorphisms (SNPs) that comprise the genome to locate ones associated with the outcome via regression models Uffelmann et al. (2021). To avoid inflation of false positives, multiple testing adjustments, such as a Bonferroni adjustment or false discovery rate (FDR) control, are carried out to the resulting *p*-values from these numerous SNP-wise hypothesis testings Benjamini and Hochberg (1995); Sesia et al. (2021); Uffelmann et al. (2021). However, after multiple testing adjustments, barely any associations are detected, especially for small-sized and medium-sized data Hong and Park (2012). The proposed solutions in literature for combatting these low-powered GWASs include only testing a subset of these SNPs given some prior knowledge, increasing *p*-value thresholds, and increasing sample size to increase the power of detecting signals Johnson et al. (2010); Uffelmann et al. (2021); Wei et al. (2009). However, the first two solutions are affected by subject opinions, and available resources determine the sample size of a study.

The advance of machine learning algorithms provides researchers with great toolkits to extract information from high-dimensional data. So, instead of looking at the marginal association between the outcome variable and each SNP separately, advanced machine learning methods can handle many SNPs simultaneously and select important ones from them while maintaining great accuracy in prediction. For example, random forest uses multiple decision trees as its base learners to combine prediction power for more accurate prediction, and it can also provide a list of SNPs ranked based on the importance of the association with the outcome variable Breiman (2001).

In this paper, we propose a novel algorithm named Bagged Logistic Regression (BLESS), which can help identify important features/SNPs when traditional SNP-wise association testing struggles. This approach leverages bootstrapping techniques to fit logistic regression models as its base learners on subsamples of the data. This enables the assessment of SNP effects within each subsample, followed by checking if the resulting *p*-values exceed a threshold after multiple testing adjustments from all subsamples. The outcome is a ranked list of SNPs based on their adjusted *p*-value. The bootstrap aggregation (bagging) approach brings new insights to the GWAS analysis.

BLESS is an ensemble algorithm that combines multiple model outputs to reduce variability and provide a more comprehensive representation of the data. In addition, BLESS can handle high-dimensional data by subsetting it into more manageable sets, which diminishes the need for extensive computational resource allocation and assists in avoiding issues of correlation and multicollinearity between SNPs. Finally, BLESS is capable of finding notable associated features even in small sample sizes, making it a convenient approach since small/medium-sized datasets are common in GWAS.

The rest of the article is organized as below. Section 2 explains materials and methods, including datasets for method illustration, the traditional SNP-wise approach, random forest, and our novel BLESS algorithm approach for GWAS. Section 3 contains a simulation study with the design and results. Section 4 is data application, where we use these methods in our ADNI datasets to identify SNPs significantly associated with the outcome of cognitive impairment. At last, Section 5 discusses the implications of the BLESS algorithm and future directions.

## 2 Materials and methods

### 2.1 Data and outcome

Data used in this article are obtained from the Alzheimer’s Disease Neuroimaging Initiative (ADNI) database (https:/adni.loni.usc.edu). The two particular datasets used are from ADNI Department of Defence (ADNI DoD) and ADNI2/Grand Opportunities (ADNI2_GO). These datasets contain rich information on genotypes, phenotypes, demographics, and cognitive assessments of 197 and 236 participants, respectively.

The outcome of interest to this study is the cognitive measure of Clinical Dementia Rating (CDR). CDR is a 5-point scale that provides a standardized way to characterize the severity of dementia, i.e., 0 = none, 0.5 = questionable, 1 = mild, 2 = moderate, and 3 = severe Hughes et al. (1982); Morris (1993). The measurement assesses a variety of cognitive domains, such as “…memory, orientation, judgment and problem-solving, community affairs, home and hobbies, and personal care” (Hughes et al., 1982; Morris (1993, 1997)). Due to a severe positive skewness of the original CDR scores in both cohorts (in ADNI DoD, kurtosis = 11.321 and skewness = 2.547; in ADNI2_GO, kurtosis = 3.294 and skewness = 1.642), the commonly used data transformation functions such as log and square root are unable to make the data symmetric. Therefore, we dichotomize the CDR scores into two categories based on a predetermined threshold of 0.5, outlined by Hughes et al. and O’Bryant et al. O’Bryant et al. (2008). That is, a person with scores <0.5 is classified as having no cognitive impairment, and a person with scores ≥0.5 has some impairment regardless of severity (mild to severe).

For the genotypes, in both cohorts, quality control (QC) is conducted by using the popular PLINK Purcell et al. (2007) software, which included removing individuals and SNPs with high percentage of missing values and low minor allele frequencies, filtering SNPs that deviated from the Hardy-Weinberg equilibrium Edwards (2008), and removing females from the data, as they were the extreme minority. Moreover, principal component analysis (PCA) of genotypes is conducted, and the top 5 principal components (PCs) are included in the model to account for the population stratification Price et al. (2006). Table 1 contains a detailed summary of the QC procedures.

**TABLE 1:** Detailed summary of the QC procedures.

Step	Description
1	Exclude SNPs and subjects with missing value rates > 0.05
2	Exclude SNPs with minor allele frequency rates < 0.05
3	Exclude SNPs with Hardy-Weinberg Equilibrium *p*-values $<1\times 10^{-6}$
4	Check sex balance. Remove females due to extreme minority
5	Principal Component Analysis. Calculating top 10 PCs to control for population stratification

The covariates included in this study are age, ethnicity, Apolipoprotein E4 (ApoE4), Mini-Mental State Examination (MMSE), Alzheimer’s Disease Assessment Scale (ADAS) and the top 5 principal components (PCs) from PCA. ApoE4 is a gene variant strongly associated with the onset of Alzheimer’s Disease (AD) Corder et al. (1993). Additionally, MMSE and ADAS are both commonly used assessments of cognitive function, particularly in the context of AD and dementia Folstein et al. (1975); Rosen et al. (1984). Note that simple imputation is used to deal with variables with a small proportion of missing values, where mode is imputed for categorical variables and mean is imputed for continuous variables. Tables 2, 3 outline the summary statistics stratified based on CDR groups for each cohort.

**TABLE 2:** Summary statistics for ADNI DoD data.

	Normal cognition	Some cognitive impairment	Total
	(N = 122)	(N = 75)	(N = 197)
Age (Years)			
Mean (SD)	69.0 ( ± 4.15)	69.5 ( ± 5.06)	69.2 ( ± 4.51)
Ethnicity (N (%))			
Not Hispanic/Latino	114 (93%)	63 (84%)	177 (90%)
Other	8 (7%)	12 (16%)	20 (10%)
ApoE4 (N (%))^a^			
Zero Alleles	88 (72%)	56 (75%)	144 (73%)
At Least One Allele	34 (28%)	19 (25%)	53 (27%)
MMSE^b^			
Mean (SD)	28.4 ( ± 1.63)	27.8 ( ± 1.83)	28.2 ( ± 1.73)
ADAS^c^			
Mean (SD)	10.3 ( ± 4.45)	13.7 ( ± 5.03)	11.6 ( ± 4.95)

^a^ ApoE4, Apolipoprotein E4.

^b^ MMSE, Mini-Mental State Examination.

^c^ ADAS, Alzheimer’s Disease Assessment Scale.

**TABLE 3:** Summary statistics for ADNI2_GO data.

	Normal cognition	Some cognitive impairment	Total
	(N = 58)	(N = 178)	(N = 236)
Age (Years)			
Mean (SD)	75.7 ( ± 5.88)	72.7 ( ± 7.32)	73.5 ( ± 3%)
Ethnicity (N (%))			
Not Hispanic/Latino	58 (100%)	171 (96%)	229 (97%)
Other	0 (0%)	7 (4%)	7 (3%)
ApoE4 (N (%))^a^			
Zero Alleles	47 (81%)	95 (53%)	142 (60%)
At Least One Allele	11 (19%)	83 (47%)	94 (40%)
MMSE^b^			
Mean (SD)	28.8 ( ± 1.44)	27.7 ( ± 2.35)	27.9 ( ± 2.21)
ADAS^c^			
Mean (SD)	10.9 ( ± 4.20)	16.4 ( ± 8.20)	15.1 ( ± 7.79)

^a^ ApoE4, Apolipoprotein E4.

^b^ MMSE, Mini-Mental State Examination.

^c^ ADAS, Alzheimer’s Disease Assessment Scale.

### 2.2 SNP-wise approach

The traditional SNP-wise approach involves applying regression models to evaluate the associations of each SNP individually to the outcome of interest. These regression models are often adjusted by various covariates. For example, logistic regression models would be used in this study, as the outcome variable is binary. These models would be adjusted by the covariates outlined in Section 2.1. From there, multiple testing adjustments must be utilized to the resulting *p*-values in order to reduce false positives Benjamini and Hochberg (1995); Uffelmann et al. (2021).

This approach suffers stringent penalties from some multiple testing adjustments and the invalid assumptions of independence among the hypotheses testing required by multiple testing procedures, leading to a lack of power in identifying important signals from small or medium-sized data. Despite these issues, this approach has the benefit of ranking the SNPs based on their marginal associations, which results in taking the top SNPs for further analysis Li (2008).

### 2.3 Random forest

Random forest is a powerful ensemble learning method that has been gaining traction due to its ability to make robust and accurate predictions. As mentioned, random forest combines the predictive power of multiple decision trees to make more accurate final predictions. By making use of bagging and random feature selection, random forest reduces model overfitting and increases generalizability to unseen data Breiman (2001).

Using the *randomForest* package in R, Liaw and Wiener (2002) we can specify the number of decision trees built as well as assess the importance of SNPs based on two measurements, mean decrease accuracy and mean decrease Gini. Mean decrease accuracy refers to the reduction in accuracy when a particular feature is permuted. Mean decrease Gini refers to the decrease in Gini impurity, which measures the probability of misclassifying the label of a randomly chosen element within a set of data points Mola and Siciliano (1997); Yuan et al. (2021). Essentially, larger values on both measures signify important features Han et al. (2016).

### 2.4 BLESS algorithm

**FIGURE 1:**
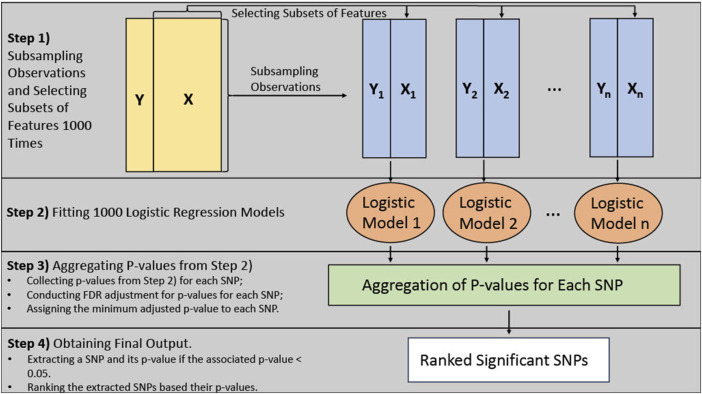
Bless algorithm architecture.

The BLESS algorithm utilizes an ensemble approach similar to random forest, where the base model is a logistic regression model instead of a decision tree. Figure 1 provides an architecture of the BLESS algorithm. The BLESS algorithm uses bootstrap subsampling without replacement and subsets the features, which randomly selects a subset of all features Pathical and Serpen (2010). These approaches can alleviate the issue of correlation and collinearity between features and improve model performance. These data subsets are then used to build logistic regression models, where the aggregated results, over multiple iterations, are subjected to FDR multiple testing adjustments, effectively creating a ranked list of features associated with the outcome.

Figure 1 demonstrates the architecture of the algorithm. In Step 1, a percentage (say 90%) of the observations and approximately the square root of the number of features (i.e., SNPs) are randomly subsampled 1000 times/iterations. Each subsample also contains the covariates of interest. For Step 2, a logistic regression model is fitted, using each of the resulting datasets from Step 1, to estimate the effects of the selected features in the dataset on the outcome variable. This process results in numerous statistical tests on SNPs through the 1000 logistic regression models. Within Step 3, we conduct the FDR multiple testing adjustments to the group of *p*-values for each SNP carried out from the previous step. We dynamically adjust the *p*-values for multiple tests by accounting for the fluctuating number of comparisons for each SNP. For example, if an SNP were selected for 100 out of the 1000 iterations, then 100 would be the number of tests for multiple testing adjustments. Finally, if any adjusted *p*-values exceed the threshold of 0.05, the corresponding SNP is identified as a significant signal and included in the final list. Moreover, among the selected SNPs, the minimum adjusted value within the group of *p*-values for that SNP is incorporated in the final list. This process aids in the creation of a ranked list based on these adjusted *p*-values in Step 4.

It should be noted the BLESS algorithm has the flexibility to increase the number of iterations. Augmenting the number of iterations can ensure each feature or genetic variant has ample opportunity to be accounted for when randomly sampling the features.

## 3 Simulation study

### 3.1 Simulation design

We carry out simulation studies to test the ability of our novel BLESS algorithm to identify the significant SNPs. We first build a blueprint model based on the ADNI DoD cohort. From the cohort, we identify the top 1000 SNPs from SNP-wise association testing based on their marginal effects. From the top 1000, we select the top 30 SNPs with their pairwise LD distances $\left(r^{2}\right)$ less than 0.8 and filter out all other SNPs with close LD distance $\left(r^{2}\geq 0.8\right)$ to any of the top 30 SNPs Mu and Zhang (2013). This results in 771 SNPs passing the filtering. In our blueprint model, the top 30 SNPs are true SNPs carrying signals, and the remaining ones do not contribute to the outcome variable. That is, our blueprint model is:

$$\text{logit}\left(\text{Pr}\left(y_{i}=1\right)\right)=\beta_{0}+\overset{771}{\underset{j=1}{\sum }}\beta_{j}{\text{SNP}}_{ij},$$

where $\beta_{0}$ is the intercept, $\beta_{j}$ is the coefficient for the $j^{th}$ SNP, i is the subject index, and j is the SNP index with i=1,2,…,n and j=1,2,…,771 . In particular, we set $\beta_{0}=0$ and generate $\beta_{j}$ by adding noise from N(mean=0.3,sd=0.1) to the true effects for j=1,2.⋯,30 and the remaining β s’ are 0. It is known that the contribution of a single SNP is small. So, for these SNPs with true signals, we chose the mean to be 0.3 and the standard deviation to be a third of the mean. This way, we can get a similar overall frequency of the outcome variable being 1 with the original data, as well as ensure the generated $\beta_{j}$ has noise different from 0. For each replication, we simulate a set of $\beta_{j}$ ’s and use the original genotyping data from the top 30 SNP to generate the outcome variables. The generated data has the same sample size as the original one (n=197) . We repeat the process 100 times to generate 100 copies of data.

For each simulated data, we apply the BLESS algorithm and obtain the list of significant SNPs for each dataset. To obtain the best combination of the tuning parameters in the BLESS algorithm, we vary the size of percentages as 80%, 90% and 100% and vary the number of selected features as 10, 30, and 50. Note that the percentages of 90% and the magic number 30 (i.e., approximately the square root of the total number of features considered) are recommended for the bagging method Liaw and Wiener (2002). Additionally, we consider increasing the number of input SNPs to 5000. Following the same setup as above, 3614 SNPs pass the filtering and are used to assess the algorithm’s performance.

We calculate the recall, precision, and accuracy for these lists of identified SNPs to assess the algorithm’s overall performance in identifying the true signals. Let true positive (TP) be the number of SNPs that BLESS can identify from the 30 blueprint SNPs, true negative (TN) be the number of SNPs that BLESS did not identify and were not in the blueprint SNPs, false positive (FP) be the number of SNPs BLESS identified and were not in the blueprint SNPs, and false negative (FN) be the number of SNPs from the blueprint SNPs that BLESS did not identify. The evaluation metrics are defined as the following.

$$\text{Recall}=\frac{\text{TP}}{\text{TP }+\text{ FN}},$$

$$\text{Precision}=\frac{\text{TP}}{\text{TP }+\text{ FP}},$$

$$\text{Accuracy}=\frac{\text{TP }+\text{ TN}}{\text{TP }+\text{ FP }+\text{ FN }+\text{ TN}}$$

As a comparison, we also carry out the SNP-wise approach to the generated data mentioned above, with adjustment of the resulting *p*-values based on an FDR adjustment. For this, the simulation metrics are set up the same as above.

### 3.2 Simulation results

**FIGURE 2:**
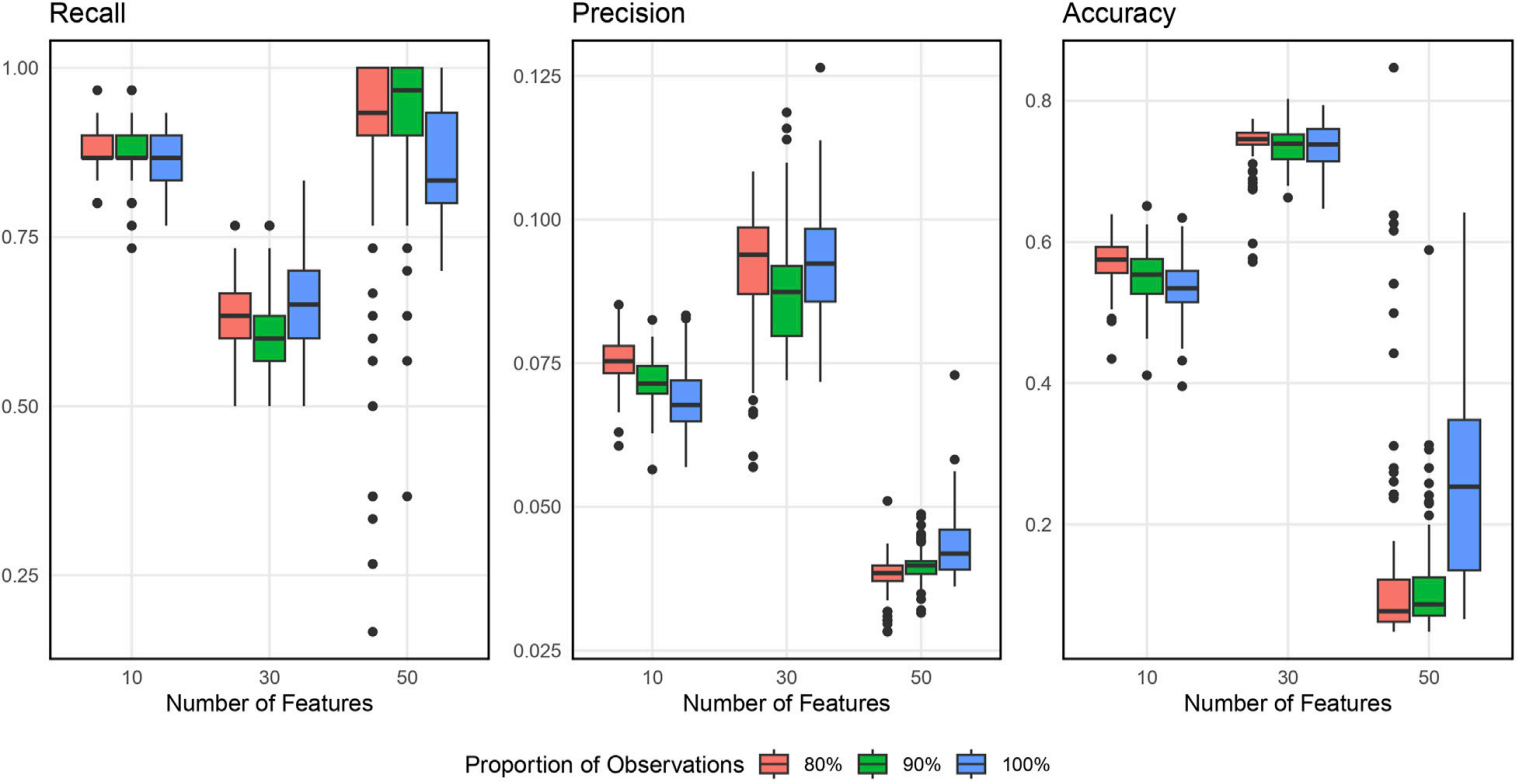
Boxplots of Evaluation Metrics from Simulation Studies for 1000 Input SNPs. The number of selected features varies as 10, 30, and 50, and the proportion of subsamples varies as 80%, 90% and 100%.

Figure 2 shows the boxplots of the resulting recall, precision, and accuracy from simulation studies across various simulation settings when inputting 1000 SNPs. Supplementary Appendix Figure S7 in Supplementary Appendix 6.2 displays the similar results when inputting 5000 SNPs. In general, the BLESS algorithm provides high recall, low precision, and medium accuracy across all the simulation settings, indicating its ability to identify most of the original blueprint SNPs accurately but also possessing quite a bit of false positive signals. The best combination of the tuning parameters is 90% with 30 (the square root of the total features) based on a balanced performance of all metrics considered.

**FIGURE 3:**
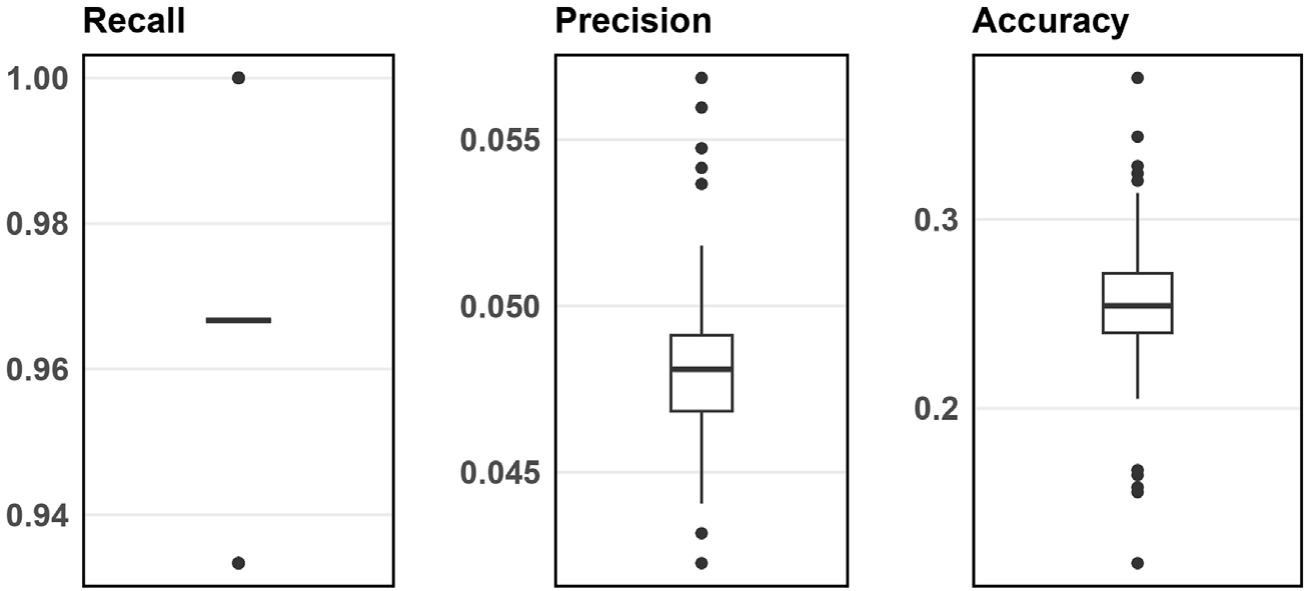
Boxplots of evaluation metrics from simulation studies for 1000 input SNPs from SNP-wise association testing.

Figure 3 displays the simulation evaluation metrics for applying SNP-wise association testing with FDR multiple testing corrections when inputting 1000 SNPs. Supplementary Appendix Figure S8 (in Supplementary Appendix 6.2) does the same but for 5000 input SNPs. As shown, the SNP-wise approach has high recall, low precision and low accuracy indicating this method can identify nearly all the blueprint SNPs, but also a large number of false signals.

From this result, BLESS generally performs better than the traditional SNP-wise approach when considering all the evaluation metrics. Now we apply the BLESS algorithm to real data.

## 4 Data application

We apply our BLESS algorithm to the two ADNI datasets introduced in Section 2.1 to demonstrate algorithm applications. For both cohorts, the outcome variable is the dichotomized CDR score indicating a normal or cognitive impairment status, and the included covariates are age, ApoE4, MMSE, ADAS, and the top 5 PCs. After the quality control, the genotyping data are included for analysis. ADNI DoD and ANDI2_GO contain the variables mentioned and the genetic information from 197 to 236 participants, respectively.

As for the analysis approach, we first apply SNP-wise association testing to both cohorts to assess their SNPs’ marginal effects associated with the binary CDR outcome. In particular, as stated previously in Section 2.2, logistic regression is used as the base model, which is adjusted by Age, MMSE scores, ADAS scores, ApoE4, Ethnicity, and the top 5 PCs, for each of the cohorts. We utilize this approach as a way to filter out the top 1000 SNPs for each group. From there, we apply both random forest and the BLESS algorithm to each filtered SNP list, comparing and contrasting the results between the two. Finally, GSEA is performed on the top 100 BLESS-identified SNPs, for ADNI DoD only, to highlight the importance of GSEA and further validate the BLESS algorithm’s ability to identify SNPs associated with cognitive impairment when traditional methods struggle.

**FIGURE 4:**
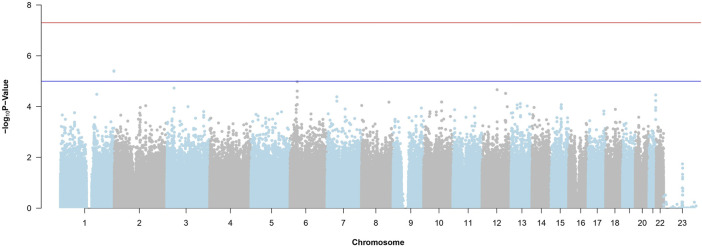
Manhattan Plot from the SNP-Wise Approach for ADNI DoD. The red horizontal line is the genome-wide significance threshold (*p*-value = $5\times 10^{-8}$ ). The blue horizontal line is the suggestive line (*p*-value = $1\times 10^{-5}$ ).

Figure 4 is the Manhattan Plot for ADNI DoD. Due to the stringent multiple testing penalty and not reaching the genome-wide threshold, no significant SNPs are uncovered. Therefore, we carry the top SNPs to use with random forest and BLESS.

### 4.1 Random forest

**FIGURE 5:**
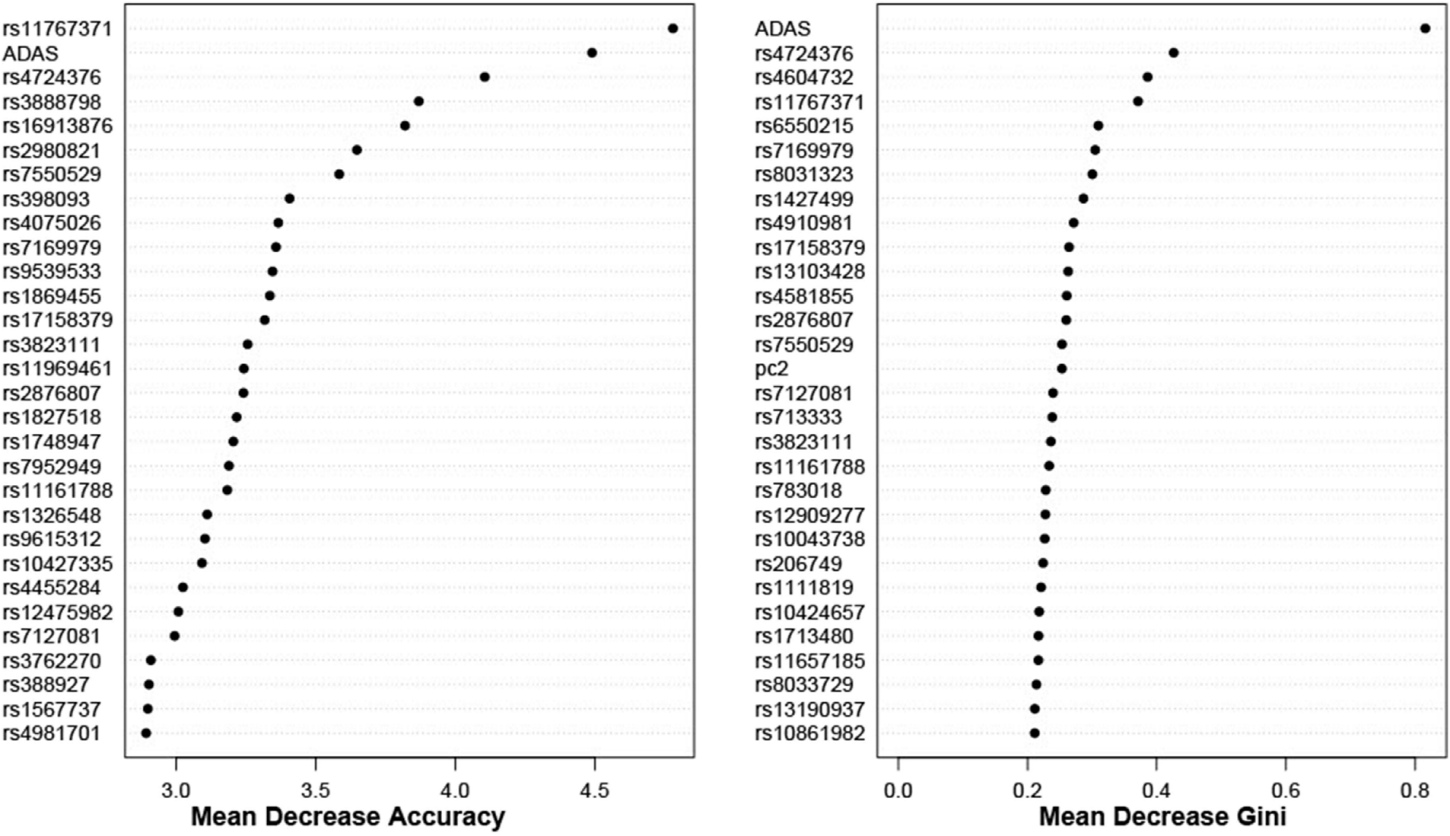
Dotchart Plot of Variable Importance from Random Forest for ADNI DoD. This model was built using 1000 trees.

**FIGURE 6:**
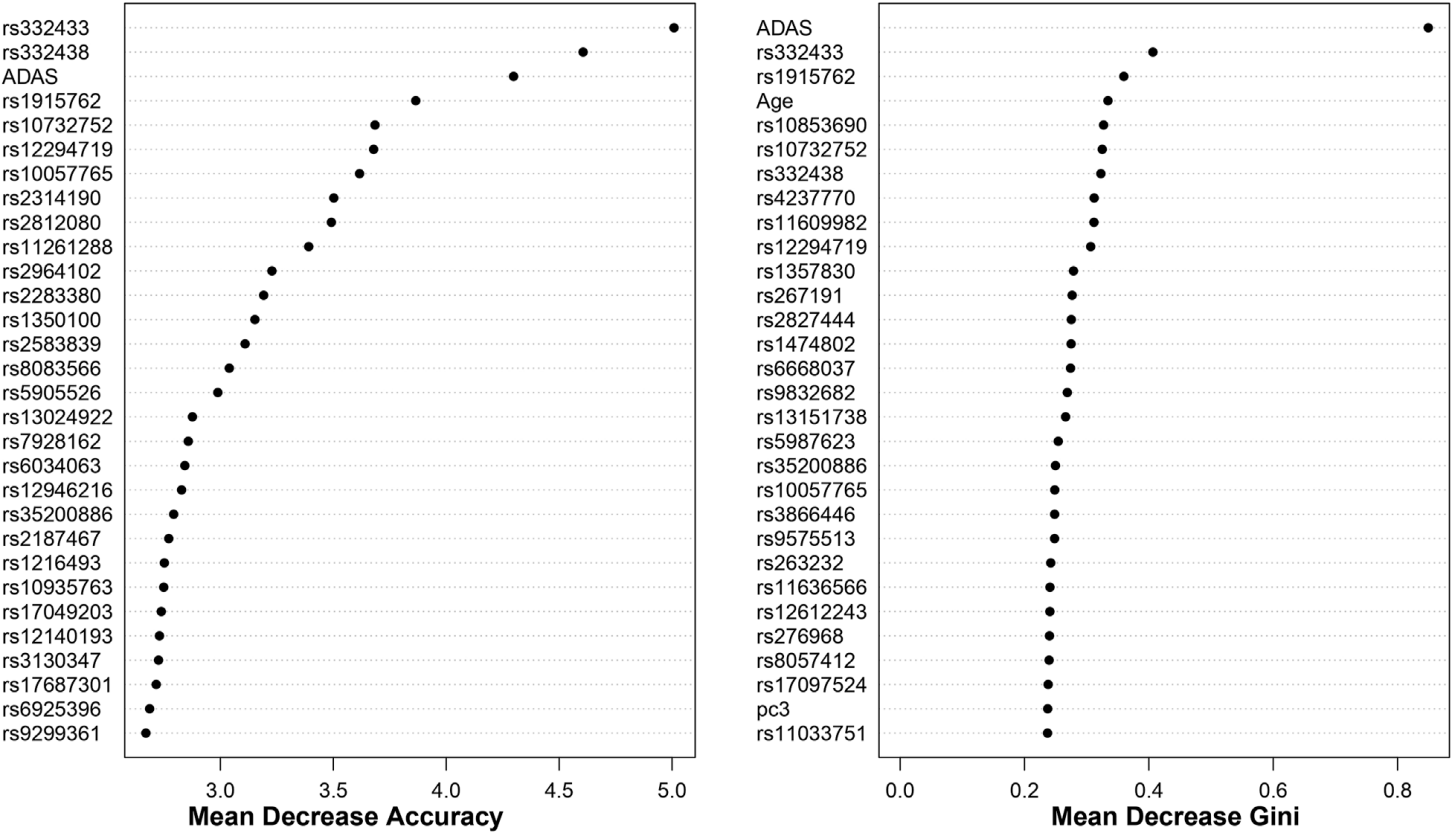
Dotchart Plot of Variable Importance from Random Forest for ADNI2_GO. This model was built using 1000 trees.

For both cohorts, using the top 1000 SNPs and the covariates indicated above, the random forest models are built with 1000 trees. The results, based on the measurements in Section 2.3, are shown in Figures 5, 6. Additionally, Appendix 6.2 contains results from building the models with 5000 trees (Supplementary Appendix Figures S9, S10).

### 4.2 BLESS results

Based on the results in Section 3.2 and for simplicity, we utilize 1000 input SNPs. Taking the top 1000 SNPs from the SNP-wise approach, in addition to the covariates and outcome variable mentioned above, we apply the BLESS algorithm to them for each cohort. As outlined in Section 2.4, we randomly sample 90% of the observations and use a subset of approximately square root the number of SNPs (about 30 SNPs) for ADNI DoD and ADNI2_GO separately, while including the covariates as fixed effects for every iteration. Using the subsample of data, we fit the logistic regression base model. We then repeat this process 1000 times and aggregate the results. From there, we apply FDR multiple testing adjustment to each grouped SNP and extract the minimum adjusted *p*-value.

Tables 4, 5 display the top 10 features identified by BLESS for their respective cohort. These top 10 BLESS identified features are accompanied by their FDR-adjusted *p*-values (FDR-p), *p*-value from SNP-wise association testing, and the rank of that feature with respect to the top 1000 SNPs based on marginal effects.

**TABLE 4:** Top 10 ranked features from the BLESS algorithm for ADNI DoD with 1000 iterations.

Feature	FDR adjusted *p*-value	Marginal *p*-value	Rank
rs10027161	< 0.001	1.105233e-03	446
rs10047112	< 0.001	7.520767e-04	291
rs10061898	< 0.001	6.580724e-04	235
rs10062726	< 0.001	1.065982e-03	430
rs10072248	< 0.001	2.012709e-03	947
rs10087406	< 0.001	3.896721e-04	134
rs10152713	< 0.001	6.389886e-04	227
rs10157147	< 0.001	3.979436e-04	137
rs10163879	< 0.001	1.284811e-04	42
rs10165591	< 0.001	1.053653e-03	425

**TABLE 5:** Top 10 ranked features from the BLESS algorithm for ADNI2_GO with 1000 iterations.

Feature	FDR adjusted *p*-value	Marginal *p*-value	Rank
rs1000960	<0.001	1.973342e-03	973
rs1001383	<0.001	1.946161e-03	961
rs10021925	<0.001	4.286250e-04	213
rs10035001	<0.001	1.467912e-03	709
rs10047855	<0.001	7.565090e-04	382
rs10050235	<0.001	1.157843e-03	547
rs10060689	<0.001	1.826003e-03	909
rs10090180	<0.001	9.629075e-04	469
rs10113	<0.001	1.549562e-03	760
rs10125807	<0.001	6.062699e-04	309

Moreover, we identify some overlap by comparing the results between random forest and BLESS. For example, in the ADNI DoD group, BLESS-identified SNPs rs11161788 (FDR-p < 0.001), rs11767371 (FDR-p < 0.001), rs17158379 (FDR-p < 0.001), rs4724376 (FDR-p < 0.001), rs7127081 (FDR-p < 0.001), rs7169979 (FDR-p < 0.001), and rs7550529 (FDR-p < 0.001) are present for both Mean Decrease Gini and Mean Decrease Accuracy in Figure 5. Similarly, from ADNI2_GO, BLESS-identified SNPs rs10732752 (FDR-p < 0.001), rs12294719 (FDR-p < 0.001), rs1915762 (FDR-p < 0.001), rs332433 (FDR-p < 0.001), rs332438 (FDR-p < 0.001), rs35200886 (FDR-p < 0.001) and rs10057765 (FDR-p = $3.12\times 10^{-2}$ ) correspond to results in Figure 6. These overlapping findings highlight the BLESS algorithms’ capacity to detect features comparable to another bootstrap aggregation method while supplying an additional set of significant features.

Additionally, as discussed in Section 2.4, the number of iterations can be increased to enhance the probability that each feature is accurately accounted for. Supplementary Appendix Tables S6, S7 in Supplementary Appendix 6.1 show the results of augmenting the number of iterations in the BLESS algorithm to 5000.

### 4.3 Post-hoc analysis

SNP to gene mapping is essential for determining the biological impact of particular SNPs. The mapping allows for a clearer understanding of how each SNP is related to the disease/trait of interest. Using the popular web-based genomic tool, g:Profiler Raudvere et al. (2019), we input the top 100 BLESS-identified SNPs for ADNI DoD with 1000 iterations, to identify which genes those SNPs land on. From there, we investigate the designated genes to identify associations with cognitive decline related to AD. An Excel file with the mapped genes can be found in Supplementary Material.

Some of the BLESS results hold promising biological implications. For example, rs10807240 is mapped to Leucine Rich Repeat And Fibronectin Type III Domain Containing 2 (LRFN2). A case study conducted by Thevenon et al. resulted in the implication of LRFN2 to deficits in a variety of tasks related to attention, working memory, and executive function Thevenon et al. (2015). Additionally, LRFN2 has been associated with antisocial personality disorder Rautiainen et al. (2016) and autism Voineagu et al. (2011).

Furthermore, these trends have been identified in mice. LRFN2-deficient mice have displayed suppressed inhibitory synapse development in the hippocampus, one of the main brain regions responsible for memory formation, and autism-like behaviours such as sensory dysfunction, impaired communication skills and social withdrawal Li et al. (2018); Morimura et al. (2019). However, despite these findings, the knowledge behind the role of LRFN2 is limited, especially for humans, and therefore requires further investigation.

Moving on from LFRN2, rs11129016 is mapped to Zinc Finger Protein 385D (ZNF385D). A GWAS conducted by Eicher et al. identified multiple SNPs, which mapped to this gene, associated with reading disability and language impairment. These disorders introduce barriers that affect communication skills development. Additionally, Eicher et al. found ZNF385D markers associated with overall brain volume Eicher et al. (2013). Brain volume changes have been associated with changes in cognition. That is, volume loss has been associated with higher rates of annual memory loss, decline in verbal fluency, and reduced attention, visuospatial ability and executive function Armstrong et al. (2020). Finally, in an ADNI study, a couple of SNPs within this gene were associated with brain arteriolosclerosis. This disease is characterized by the thickening of arterial vessel walls in the brain and has been linked to worst MMSE and CDR scores Ighodaro et al. (2016).

## 5 Conclusion and discussion

This article introduces the BLESS algorithm, an alternative approach for identifying relevant SNPs that may have been missed from the SNP-wise approach. It employs ensemble machine-learning approaches that involve subsampling and subsetting data, fitting logistic regression models for each subset, and applying FDR multiple testing adjustments to the aggregated result from multiple iterations. One of the main motivations behind this algorithm is to create an approach that can be used in GWAS when the traditional SNP-wise approach may produce inconclusive results. Additionally, as we randomly select a subset of SNPs for an iteration, the BLESS algorithm can assist in avoiding issues of correlation and collinearity or linkage disequilibrium between SNPs.

Utilizing simulation studies, we demonstrate the BLESS algorithm’s ability to identify a good proportion of the simulation-generating SNPs (i.e., true signals). From there, we apply random forest and the BLESS algorithm to data from ADNI DoD and ADNI2_GO to identify SNPs related to the cognitive measure CDR that were missed in the SNP-wise approach. Notably, we emphasize the BLESS algorithm’s ability to provide a ranked list based on FDR-adjusted *p*-values. This approach not only captures comparable results to another bagging method but also offers additional significant features.

Finally, using the top-ranked features from BLESS for ADNI DoD, GSEA was performed to explore the biological implications of the identified SNPs. This analysis serves to reinforce the application of SNP to gene mapping and provides further validation for the performance of the BLESS algorithm.

Some of the limitations behind this approach lie in the subsampling used. When using subsampling for each iteration, many features are excluded from the model-building process. Since the data is randomly subsampled, there is a potential to miss features and/or only build a few models with relevant features. However, as mentioned, the number of iterations can be increased to ensure each feature has more opportunities to be sampled. Additionally, it may be theoretically challenging to determine the optimal number of iterations. By monitoring the results between sequential iterations and looking for signs of convergence, researchers can make decisions on the stopping criteria. However, this approach is data-specific and requires researchers’ subjective opinions on the signs of convergence. Future studies can explore more robust methods for identifying the optimal number of iterations.

In conclusion, BLESS can be used to identify associated SNPs when traditional SNP-wise approaches struggle. BLESS can also handle high-dimensional data, as well as utilize common small/medium-sized data. Moreover, since this algorithm can be treated as a general ensemble machine learning method, it can be applied to data in other research sections.

## For the Alzheimer’s disease neuroimaging initiative

Data used in preparation of this article were obtained from the Alzheimer’s Disease Neuroimaging Initiative (ADNI) database (adni.loni.usc.edu). As such, the investigators within the ADNI contributed to the design and implementation of ADNI and/or provided data but did not participate in analysis or writing of this report. A complete listing of ADNI investigators can be found at: http://adni.loni.usc.edu/wpcontent/uploads/how_to_apply/ADNI_Acknowledgement_List.pdf.

## Data Availability

The data analyzed in this study is subject to the following licenses/restrictions: Authors obtained the data usage through an application process from the ADNI website. Requests to access these datasets should be directed to https://adni.loni.usc.edu/data-samples/access-data/#access_data.
