# Supplementary material for: BLESS: bagged logistic regression for biomarker identification
Source: Front Genet. 2024 Sep 10;15:1336891. doi: 10.3389/fgene.2024.1336891 (PMC11419974; doi:10.3389/fgene.2024.1336891)
Supplement: Supplementary file 1 [file Presentation1.zip › Supplement Materials/Supplymentary_Materials.pdf]

# Supplementary Material

## 1 SUPPLEMENTARY TABLES AND FIGURES

### 1.1 Figures

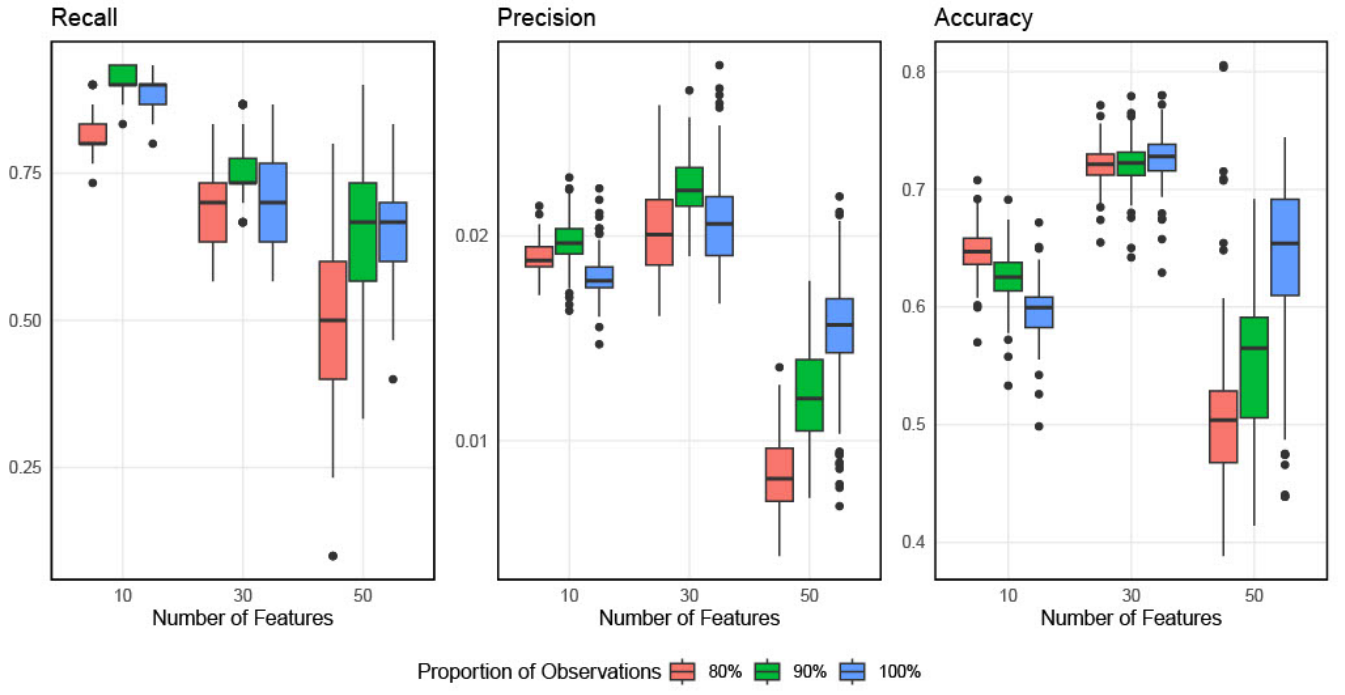


**Figure S1.** Boxplots of Evaluation Metrics from Simulation Studies for 5000 Input SNPs. The number of selected features varies as 10, 30, and 50, and the proportion of subsamples varies as 80%, 90% and 100%.

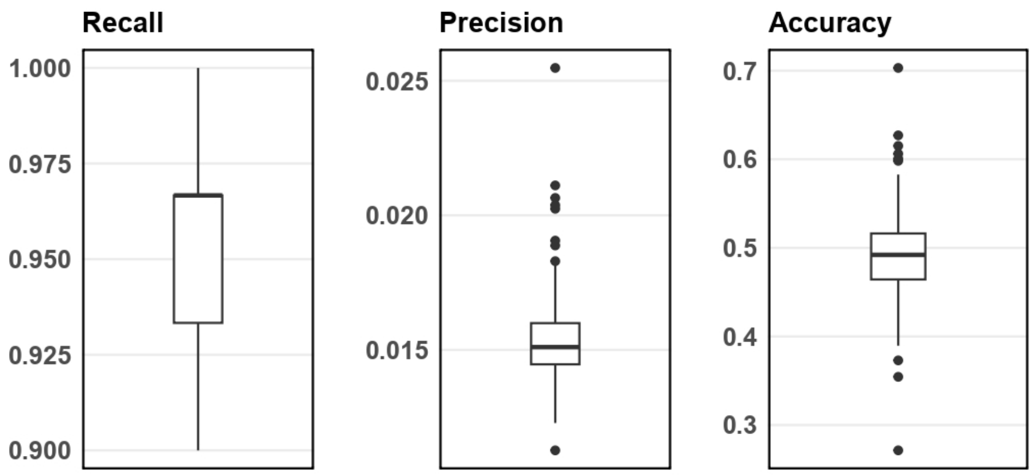


**Figure S2.** Boxplots of Evaluation Metrics from Simulation Studies for 5000 Input SNPs from SNP-Wise Association Testing.

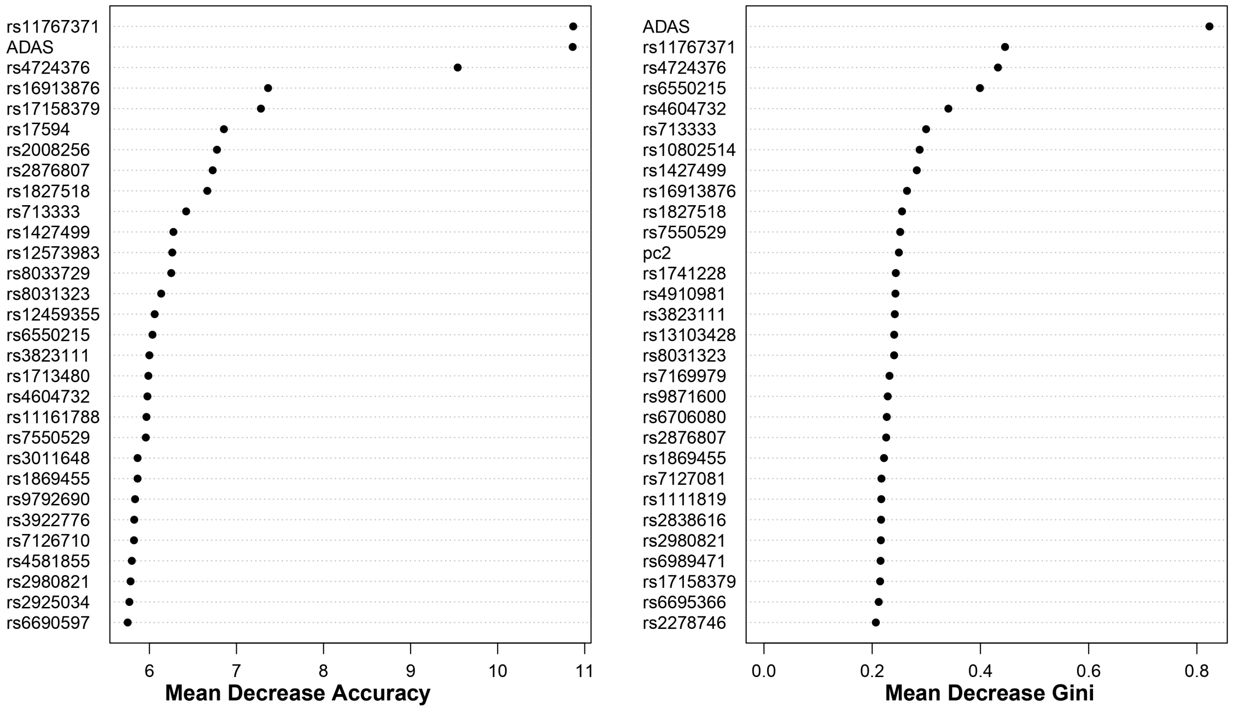


**Figure S3.** Dotchart Plot of Variable Importance from Random Forest for ADNI DoD. This model was built using 5000 trees.

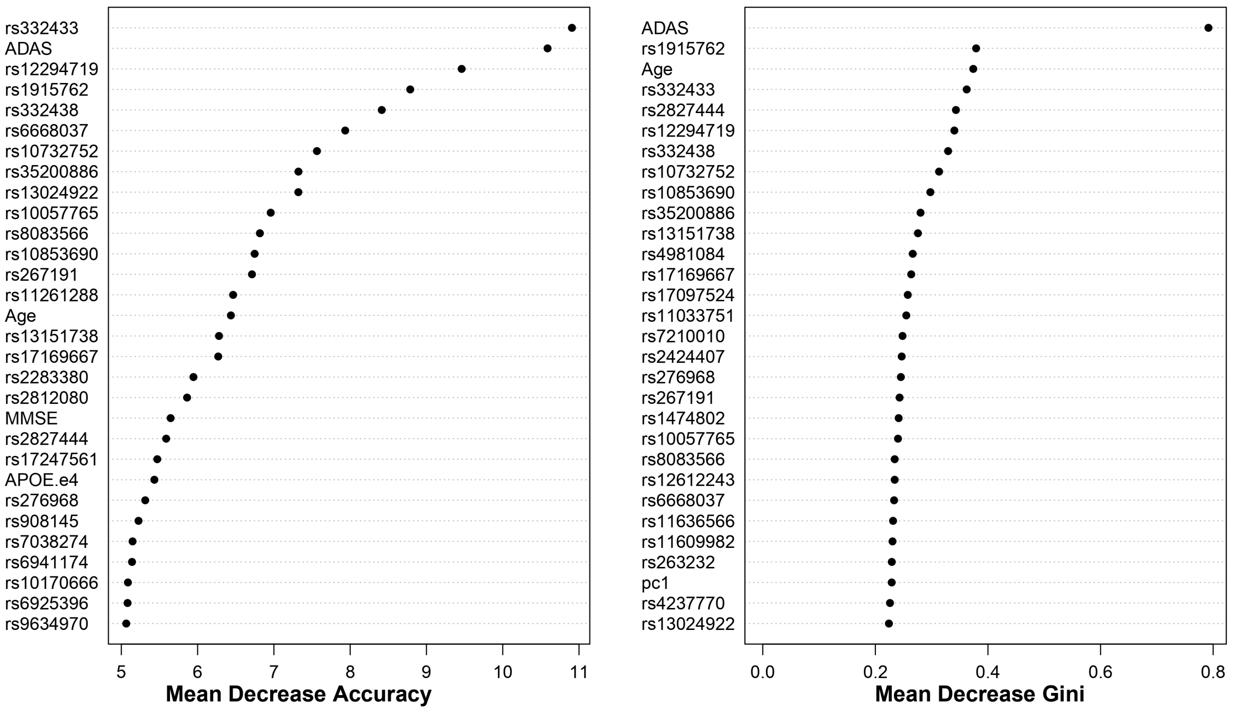


**Figure S4.** Dotchart Plot of Variable Importance from Random Forest for ADNI DoD. This model was built using 5000 trees.

## 1.2 Tables

| Feature | FDR Adjusted P-value | Marginal P-value | Rank |
|---|---|---|---|
| rs10022956 | <0.001 | 1.105233e-03 | 352 |
| rs10027161 | <0.001 | 7.520767e-04 | 446 |
| rs10043738 | <0.001 | 6.580724e-04 | 454 |
| rs10047112 | <0.001 | 1.065982e-03 | 291 |
| rs10061898 | <0.001 | 2.012709e-03 | 235 |
| rs10062726 | <0.001 | 3.896721e-04 | 430 |
| rs10072248 | <0.001 | 6.389886e-04 | 947 |
| rs10073728 | <0.001 | 3.979436e-04 | 978 |
| rs10087406 | <0.001 | 1.284811e-04 | 134 |
| rs10115613 | <0.001 | 1.053653e-03 | 77 |

**Table S1.** Top 10 Ranked Features From the BLESS Algorithm for ADNI DoD With 5000 Iterations

| Feature | FDR Adjusted P-value | Marginal P-value | Rank |
|---|---|---|---|
| rs1000821 | <0.001 | 1.679027e-03 | 820 |
| rs1000960 | <0.001 | 1.973342e-03 | 973 |
| rs1001383 | <0.001 | 1.946161e-03 | 961 |
| rs10021925 | <0.001 | 4.286250e-04 | 213 |
| rs10035001 | <0.001 | 1.467912e-03 | 709 |
| rs10047855 | <0.001 | 7.565090e-04 | 382 |
| rs10050235 | <0.001 | 1.157843e-03 | 547 |
| rs10057765 | <0.001 | 1.647422e-04 | 80 |
| rs10060689 | <0.001 | 1.826003e-03 | 909 |
| rs10090180 | <0.001 | 9.629075e-04 | 469 |

**Table S2.** Top 10 Ranked Features From the BLESS Algorithm for ADNI2_GO With 5000 Iterations

| Feature | FDR Adjusted P-value | Marginal P-value | Rank |
|---|---|---|---|
| rs10022956 | <0.001 | 1.105233e-03 | 352 |
| rs10027161 | <0.001 | 7.520767e-04 | 446 |
| rs10043738 | <0.001 | 6.580724e-04 | 454 |
| rs10047112 | <0.001 | 1.065982e-03 | 291 |
| rs10061898 | <0.001 | 2.012709e-03 | 235 |
| rs10062726 | <0.001 | 3.896721e-04 | 430 |
| rs10072248 | <0.001 | 6.389886e-04 | 947 |
| rs10073728 | <0.001 | 3.979436e-04 | 978 |
| rs10087406 | <0.001 | 1.284811e-04 | 134 |
| rs10115613 | <0.001 | 1.053653e-03 | 77 |

**Table S3.** Top 10 Ranked Features From the BLESS Algorithm for ADNI DoD With 5000 Iterations
